# Structural basis of NLR activation and innate immune signalling in plants

**DOI:** 10.1007/s00251-021-01242-5

**Published:** 2022-01-04

**Authors:** Natsumi Maruta, Hayden Burdett, Bryan Y. J. Lim, Xiahao Hu, Sneha Desa, Mohammad Kawsar Manik, Bostjan Kobe

**Affiliations:** 1 School of Chemistry and Molecular Biosciences, Institute for Molecular Bioscience and Australian Infectious Diseases Research Centre, University of Queensland, Brisbane, QLD 4072 Australia; 2 Wellcome Centre for Cell Biology, University of Edinburgh, Michael Swann Building, Max Born Crescent, Edinburgh, UK

**Keywords:** Plant innate immunity, NLR, TIR domain, CC domain, RNL, EDS1

## Abstract

Animals and plants have NLRs (nucleotide-binding leucine-rich repeat receptors) that recognize the presence of pathogens and initiate innate immune responses. In plants, there are three types of NLRs distinguished by their N-terminal domain: the CC (coiled-coil) domain NLRs, the TIR (Toll/interleukin-1 receptor) domain NLRs and the RPW8 (resistance to powdery mildew 8)-like coiled-coil domain NLRs. CC-NLRs (CNLs) and TIR-NLRs (TNLs) generally act as sensors of effectors secreted by pathogens, while RPW8-NLRs (RNLs) signal downstream of many sensor NLRs and are called helper NLRs. Recent studies have revealed three dimensional structures of a CNL (ZAR1) including its inactive, intermediate and active oligomeric state, as well as TNLs (RPP1 and ROQ1) in their active oligomeric states. Furthermore, accumulating evidence suggests that members of the family of lipase-like EDS1 (enhanced disease susceptibility 1) proteins, which are uniquely found in seed plants, play a key role in providing a link between sensor NLRs and helper NLRs during innate immune responses. Here, we summarize the implications of the plant NLR structures that provide insights into distinct mechanisms of action by the different sensor NLRs and discuss plant NLR-mediated innate immune signalling pathways involving the EDS1 family proteins and RNLs.

## Introduction

Plant pathogenic microbes pose a major threat to global crop production. To induce efficient defence responses and confer disease resistance, plants rely on two distinct types of innate immune receptors. Cell-surface pattern recognition receptors, including receptor-like kinases (RLKs) and receptor-like proteins (RLPs), perceive conserved microbial molecular structures such as pathogen-associated molecular patterns (PAMPs) and elicit defence responses known as PAMP-triggered immunity (PTI), leading to resistance to a broad range of non-adapted pathogens (Boutrot and Zipfel 2017). However, pathogens that secrete into host cells virulence factors called effectors can cause diseases by suppressing the components involved in PTI (Martel et al. 2021). Plants in turn evolved intracellular receptors, the majority of which belong to the family of nucleotide-binding leucine-rich repeat receptors (NLRs). NLRs either directly or indirectly detect effectors and initiate effector-triggered immunity (ETI) (Saur et al. 2021). ETI signalling often results in hypersensitive response (HR), a localized cell death, that restricts pathogen proliferation.

NLRs are found in both plants and animals and play pivotal roles in pathogen detection and activation of innate immune signalling, leading to programmed cell death. Plant and animal NLRs share a common prototypical structure, consisting of three domains: a variable N-terminal domain, a central nucleotide-binding domain (NBD) and a C-terminal leucine-rich repeat (LRR) domain (Jones et al. 2016). However, many plant NLRs deviate from this classic structure, having lost or acquired additional domains. The NBDs belong to the STAND (signal transduction ATPases with numerous domains) family and act as nucleotide-dependent molecular switches, with inactive ADP-bound and active ATP-bound forms (Leipe et al. 2004; Sandall et al. 2020; Song et al. 2021). Plant and animal NLRs have been proposed to have an independent origin (Urbach and Ausubel 2017). The NBDs of plant NLRs belong to a subclass named the NB-ARC domain, as they are found in the human apoptotic regulator APAF-1, plant resistance (R) proteins and *Caenorhabditis elegans* CED-4 (van der Biezen and Jones 1998). On the other hand, NLRs of most animal lineages possess the NBD of a distinct subclass of the STAND family, termed the NACHT domain, which is found in NAIP (NLR family apoptosis inhibitory protein), CIITA (MHC class II transcription activator), HET-E (incompatibility locus protein from *Podospora anserina*) and TP1 (telomerase-associated protein) (Koonin and Aravind 2000; Leipe et al. 2004). A prototypical NLR activation mechanism involves a series of conformational changes. In the absence of pathogens, the LRR domain interacts tightly with the NBD, locking it in the ADP-bound state and thus preventing NLR autoactivity (Hu et al. 2013; Wang et al. 2019b). Ligand binding to the LRR domain releases the NBD, allowing ADP-ATP exchange, and ATP binding promotes oligomerization of NLRs (Jones et al. 2016), resulting in different types of signalling assemblies, signalosomes. Examples of such NLR complexes are inflammasomes in animal cells (Martinon et al. 2002; Zhang et al. 2015) and resistosomes in plant cells (Wang et al. 2019a; Ma et al. 2020; Martin et al. 2020). The signalosomes known as apoptosomes are formed by non-NLR members of the STAND family proteins, including APAF-1, CED-4 and DARK (Zou et al. 1999; Qi et al. 2010; Zhou et al. 2015; Cheng et al. 2016, 2017). Generally, oligomerized NBD-containing proteins form ring-like structures.

Oligomerized NLRs subsequently initiate downstream signalling events, mediated by their N-terminal signalling domains. In animals, there are four subclasses of NLRs, distinguished by their N-terminal domains: NLRCs (such as NOD1, NOD2 and NLRC4), containing a CARD (caspase activation and recruitment domains); NLRPs, containing the PYD (pyrin domain); NLRBs, containing multiple BIR (baculovirus inhibitor of apoptosis protein repeat) domains; and NLRAs, containing an AD (acidic transactivating domain) (Heim et al. 2019). There are 22 and 34 NLRs in humans and mice, respectively (Pei and Dorhoi 2021). Several NLR-mediated cascades have been well-characterized. For example, activated NOD1 and NOD2 receptors recruit a kinase RIPK2 (receptor-interacting serine/threonine-protein kinase 2), through NOD^CARD^-RIPK2^CARD^ interaction, which promotes RIPK2^CARD^-RIPK2^CARD^ interaction, leading to the formation of higher-order fibrillar protein assemblies (Gong et al. 2018; Pellegrini et al. 2018). These assemblies further engage and activate MAPKs (mitogen-activated protein kinases) and transcription factor NF-κB (kappa-light-chain-enhancer of activated B-cells), leading to expression of inflammation-regulated genes and antimicrobial responses (Heim et al. 2019). Another example is an inflammasome formed by NLRP3 upon interaction with an adaptor protein ASC (apoptosis-associated speck-like protein), containing both a PYD and a CARD. The NLRP3^PYD^-ASC^PYD^ interaction promotes the recruitment of an effector pro-caspase-1 (cysteine-aspartate-specific protease 1) through ASC^CARD^-pro-caspase-1^CARD^ interaction (Lu et al. 2014; Kelley et al. 2019; Yang et al. 2019). The third example is the NAIP2:NLRC4 inflammasome formation, which is induced by a single activated protomer of NAIP2 upon ligand recognition. The oligomerized NAIP2:NLRC4 inflammasome subsequently recruits caspase-1 to its CARDs (Hu et al. 2015; Zhang et al. 2015; Wen et al. 2021). Activated caspase-1 cleaves pro-inflammatory cytokines interleukin (IL)-1β/IL-18 and thus stimulates inflammatory response (Ghayur et al. 1997). It also cleaves gasdermin D (GSDMD), which forms pores at the plasma membrane and induces lytic programmed cell death known as pyroptosis, as well as IL-1β secretion (He et al. 2015). Lastly, in the human apoptosome, APAF-1 recognizes cytochrome c and forms a heptameric complex (Zou et al. 1999; Acehan et al. 2002), which activates caspase-9, triggering apoptosis (Rodriguez and Lazebnik 1999). Although NLR oligomerization is a well-characterized activation mechanism, a new study demonstrates that NLRP3, in its inactive state, already exists as a double-ring cage structure that is necessary for the early NLRP3 activation event including trans-Golgi network dispersion (Andreeva et al. 2021).

Plant NLRs can be divided into two subfamilies, Toll interleukin-1 receptor (TIR)-NLRs (TNLs) and non-TNLs. The latter includes members with a coiled-coil (CC) motif, termed CC-NLRs (CNLs), and a resistance to powdery mildew 8 (RPW8) domain, termed RPW8-NLRs (RNLs) (Jacob et al. 2013; Shao et al. 2016; Zhong and Cheng 2016). Multiple representatives of these NLR subfamilies are present in all land plants including mosses, liverworts, conifers and flowering plants, as well as charophyte algae (Yue et al. 2012; Zhong and Cheng 2016; Gao et al. 2018), suggesting that TNL and non-TNL diversification occurred as early as in green algae (Gao et al. 2018). Compared to animal NLRs, plants (especially vascular plants) possess a remarkably diverse repertoire of NLRs (Barragan and Weigel 2021). Apart from the canonical NLR domain architecture described, many plant NLRs have accessory domains that play critical roles in effector recognition and NLR regulation. Post-LRR (PL) domains, also called C-terminal jelly-roll/Ig-like domains (C-JIDs), are uniquely found in many TNLs of flowering plants (Dodds et al. 2001; Van Ghelder and Esmenjaud 2016; Saucet et al. 2021) and directly interact with effectors, in cooperation with the LRR domains (Ma et al. 2020; Martin et al. 2020). Integrated decoy (ID) domains are present in moss and higher land plant species and mimic host proteins that are targeted by pathogen effectors (Cesari et al. 2014; Le Roux et al. 2015; Maqbool et al. 2015; Kroj et al. 2016). Furthermore, some NLRs recruit non-NLR host proteins to detect/guard effector-dependent modification of decoy proteins (Seto et al. 2017; Wang et al. 2019a, b). Plants also have dozens of truncated NLR proteins, lacking LRR and NBD motifs that can be subdivided into TX (TIR-X; without NB domain), TN (TIR-NBD) and CN (CC-NBD) families (Meyers et al. 2002, 2003), many of which have been functionally characterized in plant innate immunity (Nandety et al. 2013; Nishimura et al. 2017; Zhang et al. 2017b). As an example, in the *Arabidopsis thaliana* Columbia-0 accession genome, 160 NLR genes and 58 truncated NLR genes were identified (Meyers et al. 2003; Barragan and Weigel 2021). The number of NLRs can greatly differ within the same species and across species. Up to 251 NLR genes have been found in other accessions of *A. thaliana* (Van de Weyer et al. 2019). In rice (*Oryza sativa*), there are 445 NLRs consisting solely of non-TNL classes, as TNLs have been lost in grasses (Barragan and Weigel 2021).

Most of our current knowledge on NLR-mediated innate immune signalling comes from studies in flowering plant species. TNLs and CNLs that directly or indirectly perceive effectors are called sensor NLRs (Adachi et al. 2019b). It is increasingly evident that diverse sensor NLRs rely on a downstream network of helper NLRs, such as RNLs, as well as the NRC (NLR required for cell death) family proteins in Solanaceae species, involved in cell death execution (Wu et al. 2016, 2017; Adachi et al. 2019b; Jubic et al. 2019). In addition, gymnosperms and angiosperms have acquired lipase-like proteins that belong to the EDS1 (enhanced disease susceptibility 1) family, which are involved in a broad range of plant innate immune responses (Lapin et al. 2020). Recent studies suggest that the EDS1 family members likely relay signals from activated sensor NLRs to helper NLRs (Sun et al. 2021; Wu et al. 2021).

Structures of both CNL- and TNL-type plant NLRs have been recently elucidated, and their mechanisms of activation of downstream signalling pathways have been proposed. The first cryo-electron microscopy (EM) structures of a plant NLR come from the CNL, Arabidopsis ZAR1 (HopZ-activated resistance), including its ADP-bound inactive state, a ligand-free transition state and an ATP-bound active oligomeric state referred to as a resistosome (Wang et al. 2019a, b). Upon activation, ZAR1 can form a calcium-permeable channel at the plasma membrane through its CC domains, leading to Ca^2+^ ion flux, organelle perturbation, production of reactive oxygen species (ROS) and cell death (Bi et al. 2021). Two more recent studies uncovered cryo-EM structures of effector bound-TNL resistosomes from RPP1 (recognition of *Peronospora parasitica* 1) and ROQ1 (recognition of XopQ 1) from Arabidopsis and *Nicotiana benthamiana*, respectively (Ma et al. 2020; Martin et al. 2020). TIR domains are common components of animal innate immune signalling pathways and often function as scaffolds (Ve et al. 2015). Recent studies establish that some TIR domains of plant NLRs and of SARM1 (sterile alpha and TIR motif containing 1) involved in axon degeneration possess a nicotinamide adenine dinucleotide (NAD^+^) hydrolase (NADase) activity (Horsefield et al. 2019; Wan et al. 2019). The structures of activated RPP1 and ROQ1 resistosomes provide insights into how oligomerization of their NB-ARC domains leads to specific assembly of TIR domains to form NAD^+^-cleaving enzymes. NAD^+^ cleavage activity by plant TIR domains leads to the formation of nicotinamide and plant-specific variant cyclic ADPR (v-cADPR) (Wan et al. 2019), which potentially acts as a signal to initiate EDS1-helper NLR-mediated immune pathways to trigger cell death. In this review, we summarize the structural basis of how different classes of plant sensor NLRs recognize effectors, oligomerize and promote cell death signalling. We then discuss mechanisms of NLR-mediated innate immune signalling which requires EDS1 proteins and helper NLRs.

## The structures of ZAR1, ROQ1 and RPP1 resistosomes

ZAR1 is an ancient CNL that is highly conserved and found in many plant species (Bi et al. 2021). ZAR1 from Arabidopsis indirectly detects multiple plant pathogens using “decoy” proteins, which are targeted by pathogen effectors (Van der Hoorn and Kamoun 2008). It recognizes five distinct type III secretion effector (T3SE) families (HopZ1, HopBA1, HopF1/F2, HopO1 and HopX) from *Pseudomonas syringae* (Laflamme et al. 2020), as well as AvrAC and XopJ from *Xanthomonas campestris* and *X. perforans*, respectively (Wang et al. 2015; Schultink et al. 2019). This vast immunodiversity is mediated by ZAR1 preforming complexes with receptor-like cytoplasmic kinases (RLCKs) that belong to the ZED1-related kinases (ZRK/family XII) and recognizing effector-mediated modification of host proteins, PBS1-like kinases (PBLs/family VII) (Seto et al. 2017; Bastedo et al. 2019). For example, effectors such as HopZ1 and AvrAC acetylate or uridylylate, respectively, a decoy RLCK target and the individual targets are detected by the preformed ZAR1 complexes (Feng et al. 2012; Lewis et al. 2013).

**Fig. 1:**
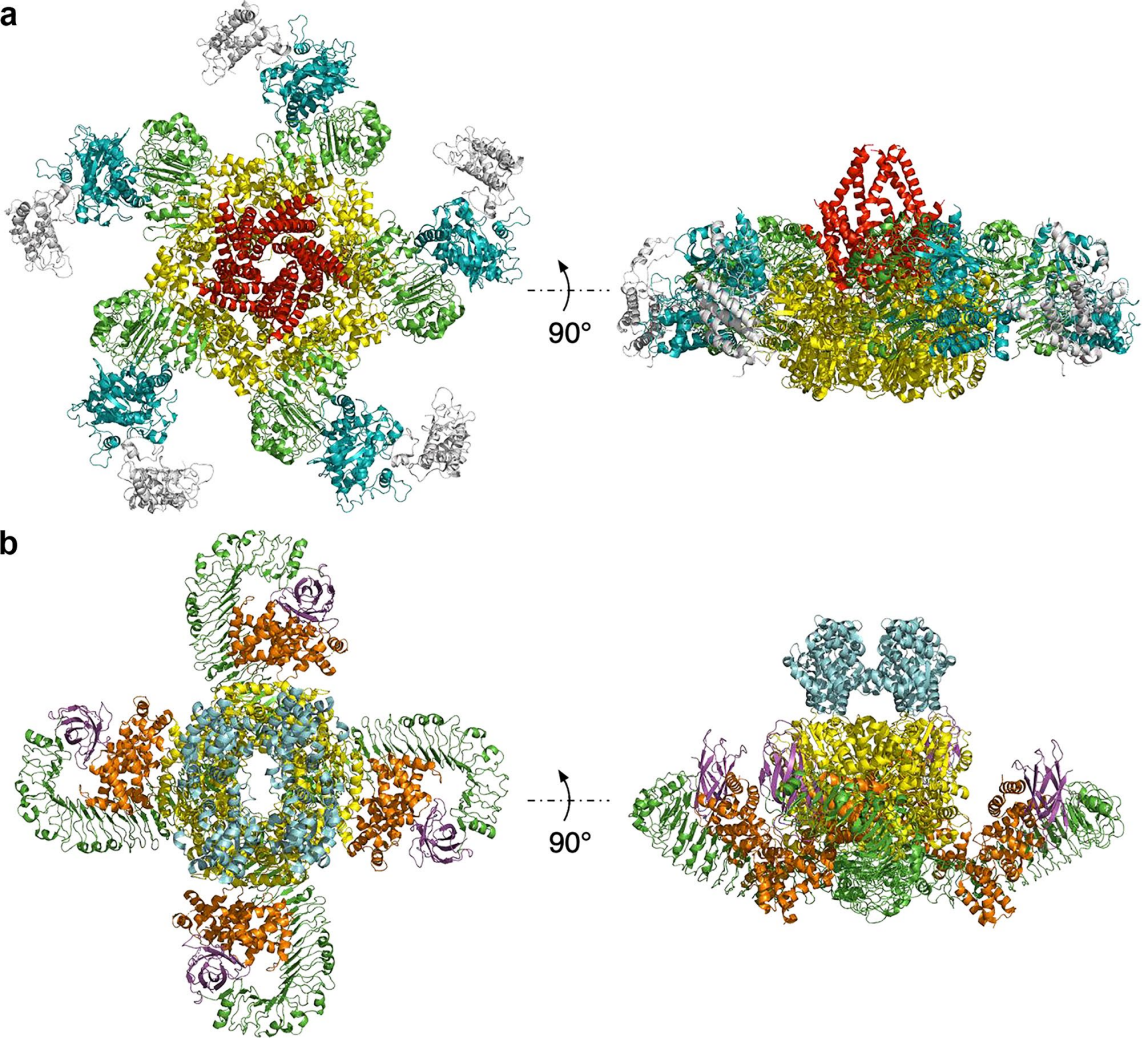
The CNL and TNL resistosomes. **a** Cryo-EM structure of the ZAR1 resistosome (PDB: 6J5T). ZAR1^CC^ is shown in red, ZAR1^NB−ARC^ in yellow, ZAR1^LRR^ in green, RKS1 in teal and PBL2^UMP^ in grey. **b** Cryo-EM structure of the RPP1 resistosome (PDB: 7CRC). RPP1^TIR^ is shown in cyan, RPP1^NB−ARC^ in yellow, RPP1^LRR^ in green, RPP1^C−JID^ in magenta and ATR1 in orange

Wang and his colleagues solved the cryo-EM structures of the inactive ZAR1:RKS1 preformed complex, the intermediate ZAR1:RKS1:PBL2^UMP^ complex and the activated ZAR1:RKS1:PBL2^UMP^ resistosome, at resolutions between 3.4 and 4.3 Å (Wang et al. 2019a, b). Prior to pathogen invasion, the inactive ADP-bound ZAR1 associates with a member of the RLCK subfamily XII-2, RKS1 (resistance-related kinase 1) pseudokinase, to form the heterodimeric complex, ZAR1:RKS1. During pathogen invasion by *X. campestris*, the effector protein AvrAC uridylylates a decoy protein of the family VII RLCK, PBL2, as well as its authentic target, BIK1 (botrytis-induced kinase 1). The uridylylated PBL2^UMP^ exclusively binds RKS1 of the preformed complex to form ZAR1:RKS1:PBL2^UMP^, which triggers ZAR1 to exchange ADP for ATP, followed by oligomerization of activated ZAR1 into a pentameric wheel-like structure (Fig. 1a). The N-terminal helices from each ZAR1 protomer protrude out of the pentameric wheel-like structure (Wang et al. 2019a). In vivo studies demonstrate that ZAR1 fused to GFP assembles into a pentamer at the plasma membrane and promotes Ca^2+^ ion influx, which likely results in direct or indirect activation of cell-death pathways (Bi et al. 2021).

TNLs such as ROQ1 and RPP1 directly recognize effectors. ROQ1 recognizes *P. syringae* HopQ1 and its close homologs XopQ from *Xanthomonas* spp. and RipB from *Ralstonia* spp. (Schultink et al. 2017; Thomas et al. 2020). The Arabidopsis *RPP1* complex locus encodes multiple RPP1 allelic variants that recognize specific variants of the effector ATR1 from the oomycete pathogen *Hyaloperonospora arabidopsidis* (Botella et al. 1998; Steinbrenner et al. 2015). This allelic variability arises in part through the co-evolution of the host and pathogen, due to the selection pressure, which the pathogen faces to evade host detection (Rehmany et al. 2005).

The structures of the full-length TNLs ROQ1, bound to XopQ, and the RPP1-WsB variant, bound to the ATR1-Emoy2 variant, were determined by cryo-EM at an overall resolution of 3.8 Å and 3.16 Å, respectively (Ma et al. 2020; Martin et al. 2020). In contrast to the pentameric ZAR1 resistosome, ROQ1 and RPP1 each form in vitro a tetrameric resistosome that resembles a four-leaf clover (Fig. 1b). ROQ1 and RPP1 directly interact with their cognate effectors through their LRR domains and additional PL domains. Effector recognition induces oligomerization of the TNLs through the NB-ARC domains, which then enables specific assembly of their TIR domains, bringing two asymmetric TIR homodimers to form NAD^+^-cleaving enzymes. The TNL resistosomes have two active sites for NAD^+^ hydrolysis, which likely facilitates downstream immune signalling, although further studies are required to establish whether these TNL resistosomes are tetramers in vivo.

## Direct and indirect effector recognition by the LRR domains

LRR domain containing proteins are found in bacteria, archaea, eukaryotes as well as viruses. The major role of LRR domains is to mediate protein–protein interactions (Kobe and Kajava 2001). A typical LRR motif is 20 to 30 amino acid long, which is tandemly arranged and contains a highly conserved segment, with the consensus LxxLxLxxN/Cx(x)L, where L is Leu, Ile, Val or Phe; N is Asn, Thr, Ser or Cys; and x is any amino acid (Kobe and Kajava 2001; Matsushima et al. 2019). The number of repeated LRR motifs in Arabidopsis NLRs ranges between 8 and 25 (Meyers 2003; Chini and Loake 2005). In other species, the LRR motif number can be much greater, reaching 47 in a lettuce NLR protein (McHale et al. 2006). LRR domains of plant NLRs display a high degree of polymorphism, compared to other domains, and are under pressure of diversifying selection (Parniske et al. 1997; Botella et al. 1998; Meyers et al. 1998; Ellis et al. 1999), shaping the NLR-effector recognition specificity over the course of plant-pathogen co-evolution. For instance, the soybean *Rps11* is a strikingly large locus and provides disease resistance to at least 12 races of *Phytophthora sojae* (Wang et al. 2021). *Rps11* contains regions encoding multiple large NLR proteins (2315–2463 amino acids) characterized by the expanded LRR motifs (Wang et al. 2021), which likely promote recognition of multiple isolates of *P. sojae*. In addition to effector recognition, LRR domains also inhibit autoactivity of plant NLRs in the absence of pathogens, through interdomain interactions, involving the N-terminal and ARC regions of the NB-ARC domain (Bendahmane et al. 2002; Moffett et al. 2002; Hwang and Williamson 2003; Rairdan and Moffett 2006; Ade et al. 2007; Qi et al. 2012; Slootweg et al. 2013).

**Fig. 2:**
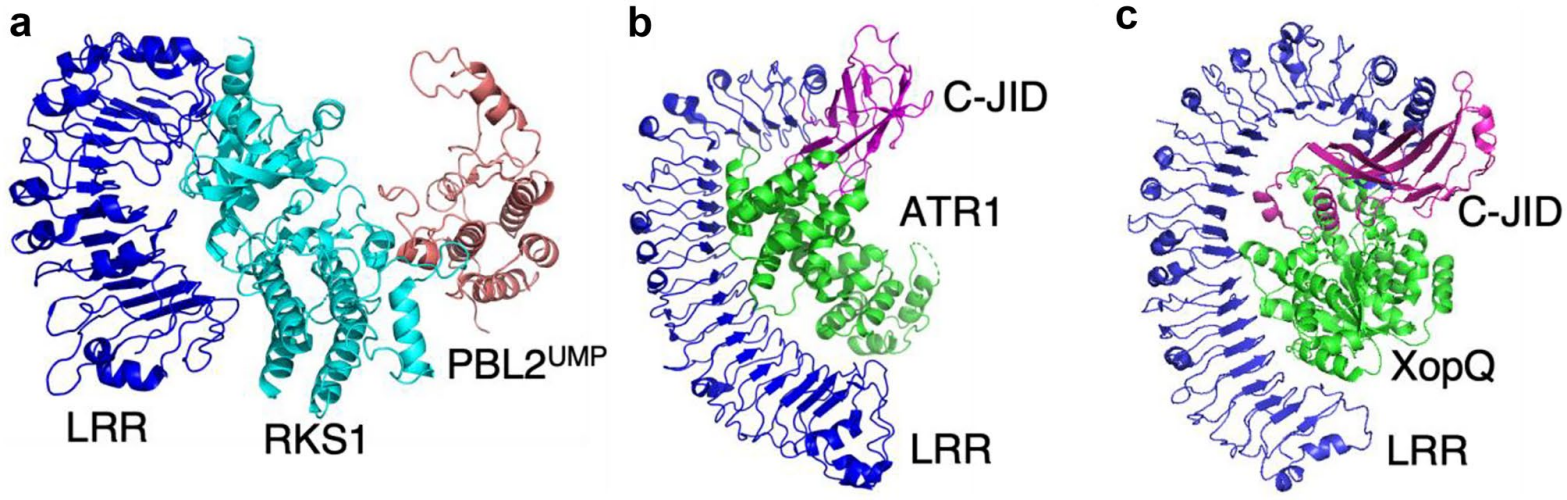
Direct or indirect effector recognition mediated by the LRRs and C-JID of the plant NLRs. **a** Indirect effector recognition by ZAR1^LRR^ (blue) pre-forming a complex with RKS1 (cyan), which interacts with the host decoy PBL2^UMP^ (red) (PDB: 6J5T). **b**, **c** Direct effector recognition by **b** RPP1 bound to ATR1 (green) (PDB: 7CRB) and **c** ROQ1 bound to XopQ (green) (PDB: 7JLU), through the LRRs (blue) and C-JID (magenta)

The three full-length plant NLR structures, ZAR1, RPP1 and ROQ1, have the characteristic horseshoe-shaped solenoid LRR domain structure, containing a variable number of LRRs: 13 repeats in ZAR1, 21 repeats in RPP1 and 24 repeats in ROQ1 (Wang et al. 2019a, b; Ma et al. 2020; Martin et al. 2020) (Fig. 2). In the ZAR1-RKS1 interaction, RKS1 contacts one of the two lateral sides of ZAR1^LRR^ (Fig. 2a) by hydrophobic interactions, in both inactive and active states, in order to facilitate indirect recognition of the effector-mediated modification of the host decoy protein, PBL2 (Wang et al. 2019a, b). Other members of the RLCK XII-2 subfamily likely engage in pre-formation of complexes with ZAR1 through their conserved residues for LRR contacts. On the other hand, RKS1 residues required for PBL2 interaction are not conserved in other members, suggesting that these contact points provide effector recognition specificities. In the inactive ADP-bound state, LRR domains keep ZAR1 in an autoinhibited form (Wang et al. 2019b). ZAR1^LRR^ makes extensive interaction with the winged helix domain (WHD, also known as the ARC2 domain) of the NB-ARC domain through the other lateral side of LRRs and packs against WHD and the helical domain (HD1, also known as the ARC1 domain). This LRR positioning of ZAR1 is different from that of animal NLRs, such as NLRC4 and APAF-1 (WD motif instead of LRRs), which rather contact the NBD (Reubold et al. 2011; Hu et al. 2013). It remains to be established whether LRR domains of other plant NLRs are oriented in a similar manner as in ZAR1 in their resting states. In the activated form, the LRR domain contributes to tight packing of the ZAR1 pentamer by interacting with HD1 of the neighbouring ZAR1 protomer.

RPP1-WsB and ROQ1 directly recognize ATR1-Emoy2 and XopQ, respectively (Ma et al. 2020; Martin et al. 2020) (Fig. 2b, c). The LRRs of RPP1 and ROQ1 bend around their cognate effector and protrude out of the resistosome, forming a clover shape tetramer, unlike ZAR1^LRR^, which contributes to tight packing of the ZAR1 pentamer (Fig. 1). RPP1^LRR^ makes direct contact with ATR1 (Fig. 2b) through hydrophobic and arginine residues at the inner concave LRR surface. Mutations in either RPP1 or ATR1 in this interface impair interaction in vitro and cell death in planta (Ma et al. 2020). These RPP1-WsB residues are not conserved among other RPP1 variants in different Arabidopsis accessions. Similarly, ATR1-Emoy2 residues involved in RPP1^LRR^ interaction are variable among ATR1 proteins in multiple *H. arabidopsidis* populations. These observations suggest that LRRs of RPP1 structurally define specific recognition of ATR1 natural variants. Similarly, ROQ1^LRR^ interacts with hydrophobic regions of XopQ (Fig. 2c), mostly through aromatic residues (Martin et al. 2020). In addition to the typical NLR^LRR^-effector interaction, ROQ1^LRR^ makes an additional contact with a conserved active site of XopQ through an extended linker between LRR23 and LRR24. This linker plays a crucial role in effector recognition, as mutations in this region are sufficient to abolish cell death.

## Diversity, structure and function of the C-terminal jelly-roll/Ig-like domains (C-JIDs) in TNLs

In addition to the prototypical three-domain architecture of plant NLRs, many TNLs including RPP1 and ROQ1 contain an additional C-terminal region following the LRR domain (Dodds et al. 2001; Meyers et al. 2002; Ma et al. 2020; Martin et al. 2020; Saucet et al. 2021). This domain, initially termed the PL domain, is characterized by four conserved signature motifs (Saucet et al. 2021). TNLs with PL domains are commonly identified in dicotyledonous plants, such as Arabidopsis, tobacco, potato, pepper, peach, flax, morning glory, soybean and grapevine, and are usually present in more than 50% of the total number of TNLs in a species (Dodds et al. 2001; Van Ghelder and Esmenjaud 2016; Van Ghelder et al. 2019; Ma et al. 2020; Martin et al. 2020; Saucet et al. 2021). Structural analyses of RPP1 and ROQ1 resistosomes uncovered that these domains form immunoglobulin-like and jelly-roll folds, containing, respectively, eight and nine antiparallel β-strands, which form two β-sheets and fold into a β-sandwich (Ma et al. 2020; Martin et al. 2020) (Fig. 2b, c). Due to the established structures, hereafter, the PL domain is referred to as the C-JID.

The C-JIDs of RPP1 and ROQ1 are involved in direct interaction with their effectors. The C-JID: effector interaction is equivalent to the complementary binding between an antibody and an antigen (Martin et al. 2020). While RPP1^C−JID^ and ROQ1^C−JID^ share a similar β-sandwich core, their loop regions that interact with the effectors are different (Martin et al. 2020). In RPP1^C−JID^, a loop between the 7th and 8th β-strands and a region involving the 4th and 5th β-strands interact with ATR1 through hydrogen bonds and hydrophobic interactions (Ma et al. 2020). Multiple allelic variants of RPP1 display polymorphism within these loops (Ma et al. 2020), likely contributing to effector recognition specificity. The RPP1^C−JID^ loop contacts an aspartic acid of ATR1, although this residue is only found in a few ATR1 allelic variants (ATR1-Emoy2, ATR1-Maks9, ATR1-Emco5) (Ma et al. 2020). Substitution of aspartic acid to tyrosine, which naturally occurs in ATR1 variants (ATR1-Cala2, ATR1-Emwa1) not recognizable by RPP1-WsB, abolishes RPP1-ATR1 interaction in vitro (Ma et al. 2020). In ROQ1^C−JID^, there are two loops connecting β-strands that are responsible for XopQ detection. A loop between the 7th and 8th β-strands, similar to RPP1^C−JID^, is crucial for effector interaction (Martin et al. 2020). Furthermore, the unique feature of ROQ1^C−JID^ that contributes the most to its ligand binding is a 33-residue loop between the 3rd and 4th β-strands, termed the NR loop, found in several closely related species of tobacco. Hydrophobic side chains of the NR loop bind the conserved residues within the active site of XopQ for ADPR binding. Mutations in either loop region of ROQ1^C−JID^ attenuate HR (Ma et al. 2020).

The C-JID consists of approximately 150 amino acids, with four conserved signature motifs (Saucet et al. 2021). According to Saucet and colleagues (2021), the first motif is located immediately after the LRR domain, with the consensus P-X-[Y/E/W]-F. The second motif contains a conserved cysteine residue and subsequent hydrophobic residues. The third and fourth motifs involve a conserved histidine residue and Cys-Gly residues, respectively. In many cases, the C-JID is followed by a nuclear localization signal, transmembrane domain or other regions of unknown functions (Saucet et al. 2021). Although seemingly rare, some TNLs contain multiple C-JID sequences. The *TNL1* gene from Myrobalan plum (*Prunus cerasifera*), also known as the *Ma* resistance gene, encodes a TNL containing five C-JID motifs (Claverie et al. 2011). There are also 12 *TNLs* in the peach genome that encode more than one C-JID (Van Ghelder and Esmenjaud 2016). Functional analyses previously demonstrated that truncation of the C-JID of flax *P2* resulted in loss of disease resistance (Dodds et al. 2001). Furthermore, upon deletion or mutations in the conserved C-JID sequences, the Arabidopsis TNL RPS4 (resistance to *P. syringae* 4) as well as the tobacco TNL N failed to trigger cell death in *N. benthamiana* (Saucet et al. 2021). Therefore, C-JID is indispensable for TNL-mediated innate immunity. The versatile *Ma* gene, which has five C-JID sequences, confers a complete spectrum of disease resistance to more than 30 species and isolates of root knot nematodes (Claverie et al. 2011). It is tempting to speculate that these domains are the key factors determining recognition of multiple nematode effectors by the *Ma* receptor.

Some TNLs, encoded by genes that are clustered in a head-to-head orientation in genomes, often function as pairs with roles as “sensor” for effector recognition or “executor” for signalling. Interestingly, analysis of Arabidopsis paired TNLs revealed that the conserved C-JID sequences are found in executor TNLs, rather than sensor TNLs, which have degenerated C-JIDs (Saucet et al. 2021). In the RRS1:RPS4 (sensor/executor) pair, RPS4^C−JID^ is involved in the regulation of RPS4 to maintain its inactive state (Saucet et al. 2021), suggesting that C-JID may have non-sensory functions in addition to effector recognition (Saucet et al. 2021).

In summary, structural analyses of activated RPP1 and ROQ1 provide insights into how direct effector recognition specificity is achieved through multiple interfaces, involving LRR and C-JID regions. More research of various NLR structures is necessary to elucidate specific mechanisms of direct and indirect effector recognition for specific NLR-effector/guard/decoy pairs. Such structural information should guide us to improve engineering of NLRs that precisely target effectors, as so far successful implementation of modified NLRs has been limited (Tamborski and Krasileva 2020).

## ATP-dependent activation and oligomerization through the NB-ARC domain

**Fig. 3:**
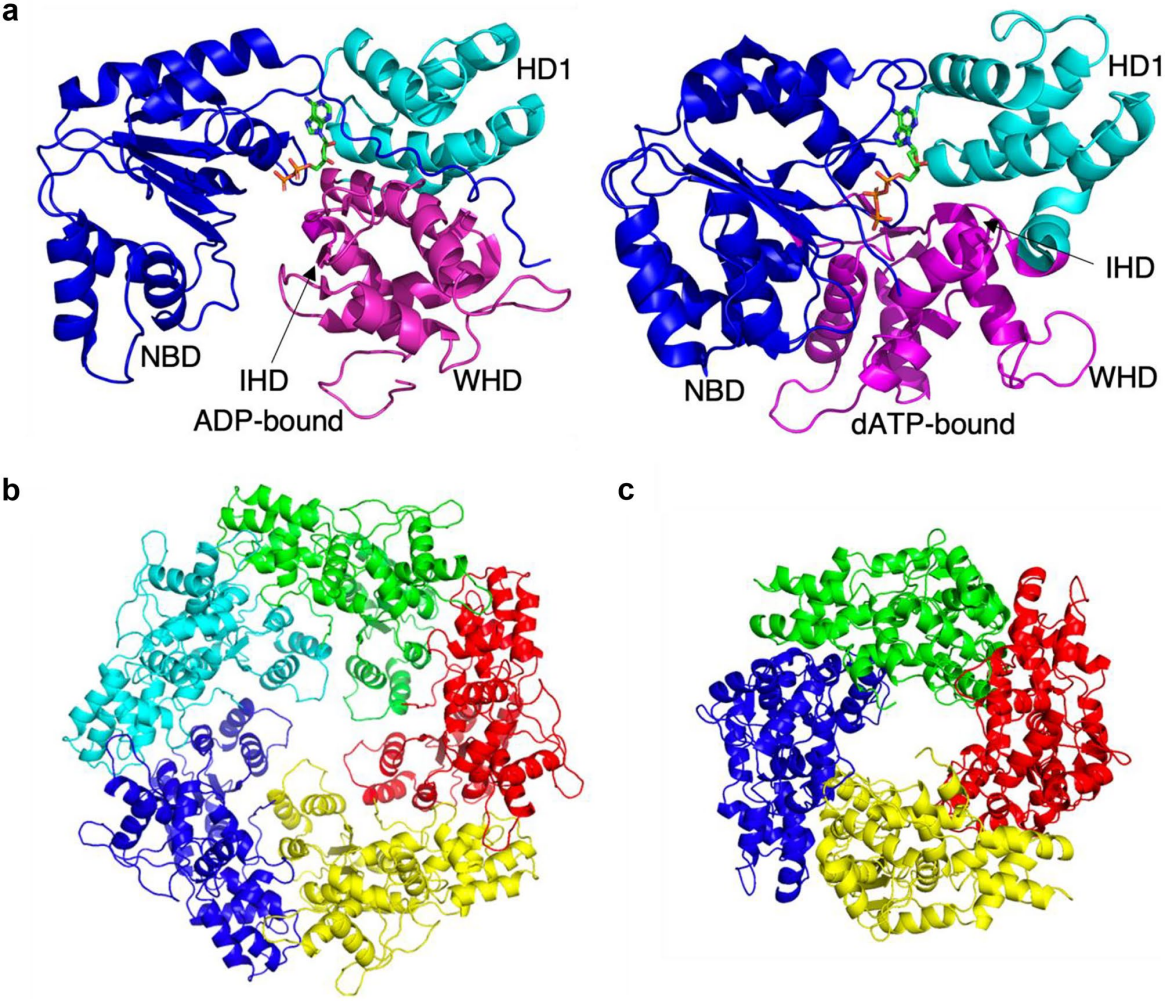
The structure of the ZAR1 NB-ARC domain. **a** The ZAR1 NB-ARC domain in its ADP-bound form (left, PDB: 6J5W) and dATP-bound form (right, PDB: 6J5T). NBD (blue), HD1 (cyan) and WHD (magenta) are shown. The MHD motif (IHD in ZAR1) is repositioned upon ADP-ATP exchange. **b**, **c** The NB-ARC domain of **b** ZAR1 (left, PDB: 6J5W) and **c** RPP1 (right, PDB: 7CRC) resistosomes. Different colours represent individual protomers

The central NB-ARC domain, consisting of NBD, HD1 and WHD, functions as a molecular switch that regulates NLR activity by binding adenosine nucleotides, ADP or ATP (Wang et al. 2019a; Ma et al. 2020; Martin et al. 2020) (Fig. 3a). Inactive NLRs are in an ADP-bound state, as they likely have either a binding preference for ADP over ATP or an intrinsic property for ATP hydrolysis (Tameling et al. 2006). Plant NLRs possess the conserved Walker-B motif in NBD, which is crucial for ATP hydrolysis (Pan et al. 2000; Meyers et al. 2003). Similar to APAF-1 (Reubold et al. 2009), RPP1 was shown to exhibit greater ATPase activity in its catalytically inactive form than effector-activated form (Ma et al. 2020), supporting its intrinsic ATPase activity. In a resting ADP-bound form, NLRs maintain their autoinhibited conformation by having HD1, NBD and WHD in a closed state (Riedl et al. 2005; Reubold et al. 2011; Hu et al. 2013; Wang et al. 2019b) (Fig. 3a). WHD contains a three-residue motif known as the MHD motif, which is necessary for ADP binding, and therefore the positioning of WHD influences the nucleotide binding (Fig. 3a). In the inactive state, ZAR1^WHD^ interacts with the β-phosphate group of ADP by a hydrogen bond (Wang et al. 2019a), bringing WHD close to NBD (Riedl et al. 2005). The MHD motif has been well characterized among plant NLRs, and mutations lead to effector-independent cell death activation, likely due to removal of autoinhibitory NBD-WHD interactions (van der Biezen and Jones 1998; Tameling et al. 2006; van Ooijen et al. 2008; Bernoux et al. 2011, 2016; Williams et al. 2011).

Upon pathogen detection, the autoinhibitory interactions within the NB-ARC domain of ZAR1 are disrupted by repositioning of WHD away from the ADP binding pocket, followed by ADP-ATP exchange and oligomerization of NLR protomers through the NB-ARC domain (Fig. 3a). NBD and HD1 stabilize the bound ATP molecule. Residues in the conserved P-loop motif in NBD mediate interaction with the β-phosphate moiety of ATP (Wang et al. 2019a; Martin et al. 2020). The Walker-B motif of NBD is involved in coordination of a cofactor, Mg^2+^ and ATP hydrolysis (Bentham et al. 2017; Martin et al. 2020). Oligomerization is mediated by two major interfaces, an NBD-NBD interface and a HD1-WHD interface, formed by adjacent NLR protomers.

Both plant and animal NLRs form wheel-like structures: the pentameric ZAR1 resistosome (Wang et al. 2019a), the tetrameric RPP1 and ROQ1 resistosomes (Ma et al. 2020; Martin et al. 2020), an octameric *C. elegans* CED-4 apoptosome (Qi et al. 2010), a heptameric APAF-1 apoptosome (Acehan et al. 2002; Zhou et al. 2015), an octameric DARK (Drosophila APAF-1 related killer) apoptosome (Cheng et al. 2017) and an undecameric NAIP2:NLRC4 inflammasome (Zhang et al. 2015). The NBD, HD1 and WHD are similarly packed to stabilize the oligomeric forms of these NLRs (Fig. 3b, c). Compared to apoptosomes and inflammasomes, plant NLR resistosomes characterized to date have a more compact conformation. Oligomerization of NLRs appears to require N-terminal linker residues of NBD domains. For example, in the NLRC4 inflammasome, this N-terminal region forms an α-helix structure, which likely allows larger oligomer assembly (Martin et al. 2020). In comparison, plant NLRs have the N-terminal loop instead of any secondary structure (Wang et al. 2019a; Martin et al. 2020). This loop region links NBD with adjacent subunits more strongly to each other, allowing for the less steric hindrance to pack more tightly (Wang et al. 2019a; Martin et al. 2020). Therefore, the N-terminal linker region may contribute to determining the number of monomers in the oligomer (Martin et al. 2020). Alterations of the region involving these linkers, by substitutions, deletions or swaps among Arabidopsis CNLs, affect their ability to trigger cell death (Wroblewski et al. 2018).

Based on current models, ATP binding by NB-ARC domains results in the formation of activated plant NLRs resistosomes. However, the RPP1 resistosome structure shows an ADP molecule, instead of ATP, bound to the P-loop (Ma et al. 2020). In order to bind ATP, NLRs appear to require an arginine residue within a conserved T-T/S-R motif (also known as the sensor-1 motif) to make a contact with the γ-phosphate group of ATP (Proell et al. 2008). Animal NLRs and plant CNLs have the highly conserved arginine residue, whereas plant TNLs including RPP1 often have a differently charged (Glu) or polar (Gln) residue (Ma et al. 2020). Despite ADP binding, which could potentially destabilize resistosomes, these TNLs likely maintain their stable oligomeric states through acquisition of additional interactions. In support of this view, the RPP1 resistosome is stabilized by an additional β2-α2 loop that promotes interaction between NBD of one protomer and WHD of another (Ma et al. 2020). On the other hand, ROQ1 and ZAR1, which possess the conserved arginine that binds ATP in the resistosomes, seem to have a shorter β2-α2 loop, suggesting that such a loop is not required for stabilization of the ATP-bound form (Ma et al. 2020). These observations suggest that activated NLR resistosomes can be maintained by either ATP or ADP binding.

## The CC domains of the ZAR1 resistosome are proposed to form a calcium influx channel

**Fig. 4:**
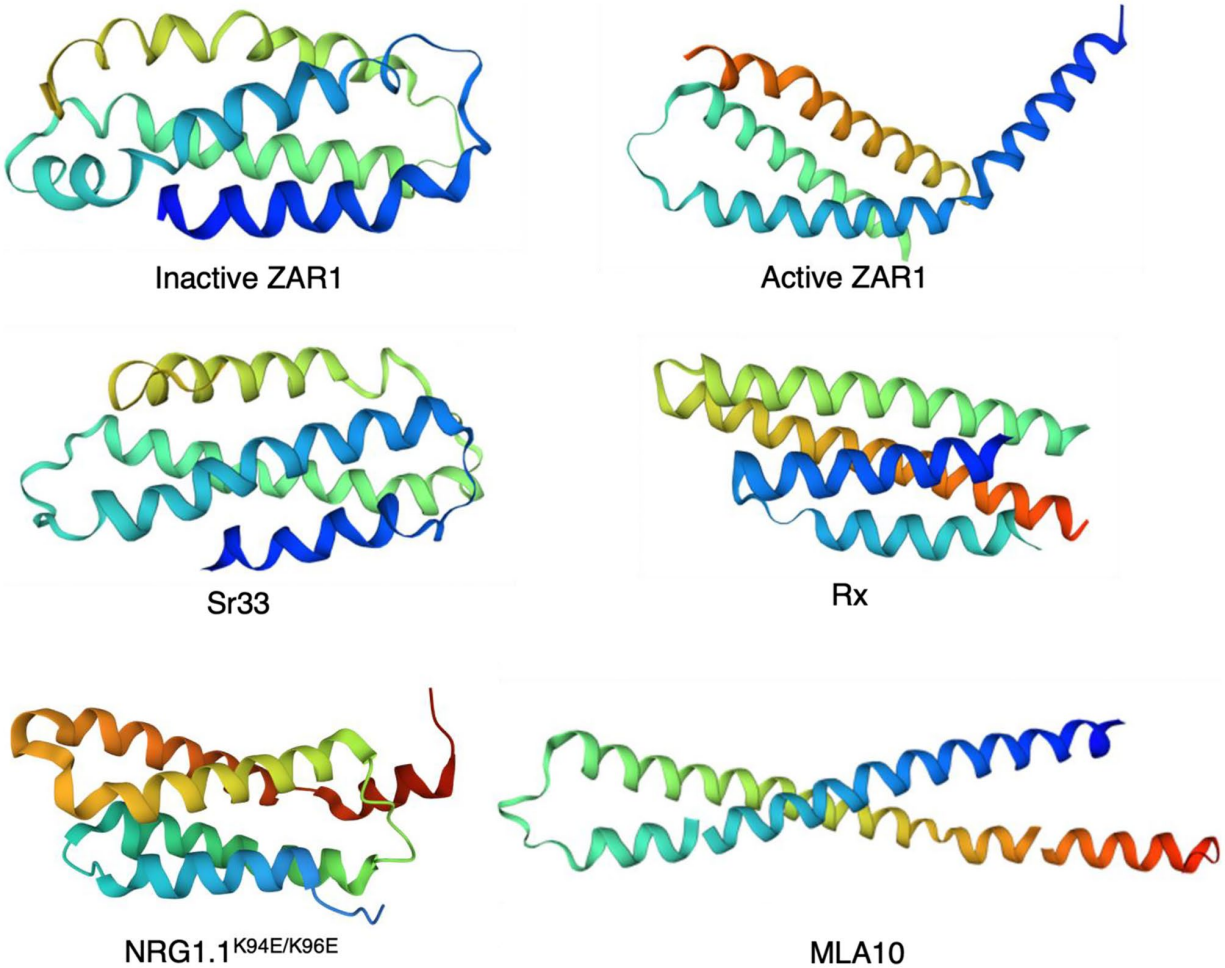
Structures of CC and CC_R_ domains from plant NLRs. Inactive Arabidopsis ZAR1 (residues 1–113, PDB: 6J5W), active Arabidopsis ZAR1 (residues 4–138, PDB: 6J5T), wheat Sr33 (residues 6–120, PDB: 2NCG), potato Rx (residues 1–112, PDB: 4M70), Arabidopsis NRG1.1 K94E/K96E mutant (residues 1-124, PDB: 7L7W) and barley MLA10 (residues 5–120, PDB: 5T1Y)

To date, the available structures of CC and CC_R_ domains of plant NLRs include crystal structures of barley MLA10^CC^ (residues 5–120), potato Rx^CC^ (residues 1–112) and Arabidopsis NRG1.1^CCR^ (N required gene 1; residues 1–124), a solution 3D structure of wheat Sr33^CC^ (residues 6–120) and cryo-EM structures of full-length ZAR1, including inactive and active states (Maekawa et al. 2011; Hao et al. 2013; Casey et al. 2016; Wang et al. 2019a, b; Jacob et al. 2021) (Fig. 4). All known CC or CC_R_ domain structures display compact four-helical bundles, except for MLA10^CC^, in which the monomer has a helix-loop-helix structure with a long rod shape and forms an intertwined homodimer (Maekawa et al. 2011). This fragment of MLA10^CC^ is monomeric in solution and lacks the C-terminal residues necessary for self-association and HR in planta (Casey et al. 2016). The MLA10^CC^ dimer structure may reflect the dynamic CC domain conformation, resulting from the release of the N-terminal α1 helix. In ZAR1^CC^, the amphipathic α1 helix is solvent-exposed upon activation, forming a funnel-shaped structure by hydrophobic and electrostatic interactions with an α1 helix of adjacent monomer. By contrast, the α1 helix of MLA10^CC^ is largely buried by the formation of the dimer through interacting with the α3 helix. Unlike the activated ZAR1, the α4 helix of MLA10^CC^ also extends out. Hence, the MLA10^CC^ dimer structure does not appear to be physiologically relevant.

In an inactive state, ZAR1 adopts the typical four-helix bundle conformation, where an α1 helix is buried and interacts with LRR and WHD. Transition from inactive to active ZAR1 is suggested to involve a sequence of structural rearrangements: (1) movement of WHD away from NBD, (2) ADP release and subsequent ATP binding, (3) rearrangement of the N-terminal α1 helix, which protrudes out towards the surface, and (4) oligomerization of ZAR1 through NBD. The resulting pentameric ZAR1 resistosome displays an α-helical barrel structure with pore formed through the CC domains (Wang et al. 2019a). Activation of ZAR1 also involves formation of a long helix within the CC domain (α4B; residues 108–138), through incorporation of a flexible region (residues 89–111), instead of a helix seen in inactive ZAR1 (α4A; residues 89–111) (Wang et al. 2019a). The crystal structure of the CC_R_ domain of NRG1.1 is analogous to that of the four-helix bundle CC domain structures as well as the animal mixed-lineage kinase-like (MLKL) protein which functions as a cation channel (Jacob et al. 2021). The NRG1.1 CC_R_ domain possesses an N-terminal flexible fragment (residues 1–16), which possibly contributes to pore formation upon activation in a similar way as the ZAR1 α1 helix. While the deletion (Δ16) allows NRG1.1 oligomerization, it results in loss of Ca^2+^ influx (Jacob et al. 2021).

Consistent with the cryo-EM structure, in vivo analyses reveal that the activated ZAR1 can oligomerize into a pentamer and exhibit a selective Ca^2+^ cation channel activity (Hu et al. 2020; Bi et al. 2021). The influx of calcium requires the negatively charged carboxylate rings involving the conserved Glu-11 residue. The ZAR1 resistosome localizes to the plasma membrane, although other membranous structures are not excluded (Bi et al. 2021). Hypothetically, the ZAR1-dependent cell death and immune signalling mechanism could involve effector-mediated activation of ZAR1 that leads to calcium ion influx, followed by perturbation of organelles such as vacuoles and chloroplasts, production of reactive oxygen species (ROS), disintegration of the nucleus, disruption of the plasma membrane integrity and subsequent cell rupture (Bi et al. 2021). However, while activated ZAR1 and Ca^2+^ influx are essential for the cell death triggering, the exact mechanism of the cell death remains to be established (Bi et al. 2021). Another recent study demonstrated that the auto-active RNL NRG1.1 leads to higher-order complex formation in the plasma membrane puncta and facilitates calcium influx by formation of cation channels, in both plant and human cells (Jacob et al. 2021). The auto-active NRG1.1 forms a non-selective cation channel, which is permeable to Ca^2+^ (Jacob et al. 2021). Both RNL subfamily members, NRG1 and ADR1 (activated disease resistance 1), retain the conserved negatively charged N-terminal residues and their mutations attenuate Ca^2+^ ion influx and cell death (Jacob et al. 2021).

## Tetrameric assembly of the TIR domains facilitates NADase activity

The TIR domain is the catalytic signalling domain of TNLs. The TIR domain is both necessary and sufficient for induction of HR, as when expressed alone, it is capable of inducing cell death in plant tissue (Bernoux et al. 2011). Plant TIR domains display structural similarity to TIR domains from animals and bacteria. The overall tertiary structure is comprised of a central β-sheet core, surrounded by loops and α-helices, in a flavodoxin-like fold (Ve et al. 2015). The helices and loops mediate homotypic interactions with other TIR domains. A subset of TIR domains from animals, plants and bacteria are now understood to have NAD^+^ hydrolase (NADase) activity, able to cleave NAD^+^ into ADPR and nicotinamide, and, in the case of plant and bacterial TIRs, a yet unknown variant of cyclic ADPR (v-cADPR) (Wan et al. 2019). Self-association of TIR domains is essential for activation of NAD^+^ hydrolysis activity by plant TIR domains and the mammalian TIR-containing NADase, SARM1, which is involved in axon degeneration (Horsefield et al. 2019; Wan et al. 2019). X-ray crystallographic and recent cryo-EM studies have greatly enhanced our understanding of how TIR domains function in plant innate immunity and how they are regulated, activated and arranged in the context of oligomeric resistosomes.

**Fig. 5:**
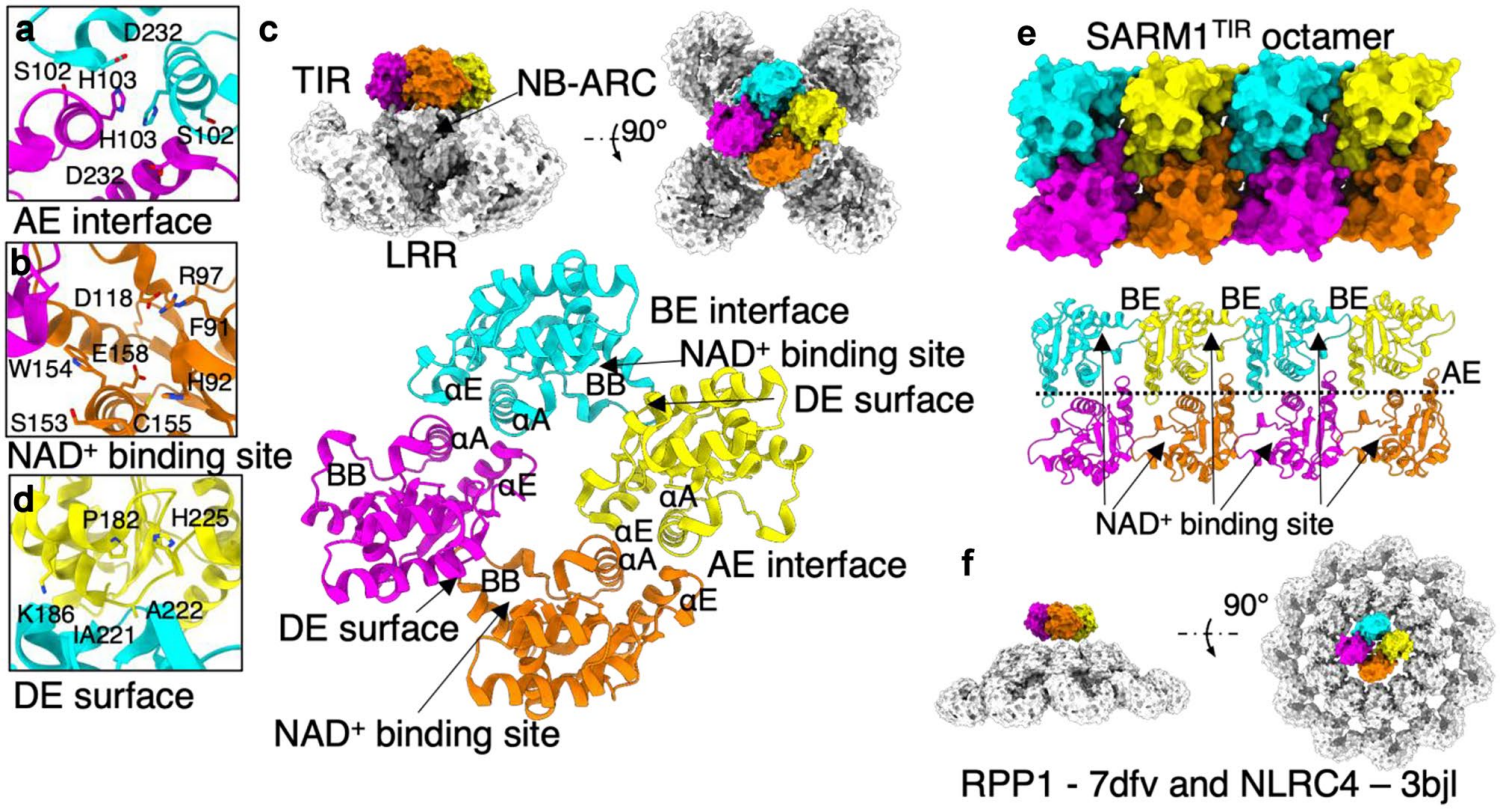
Assembly of plant TIR domains in the activated NLR resistosome. **a** The AE interface of the RPP1^TIR^ tetramer, with important conserved residues shown (PDB: 7DFV). **b** The NAD^+^ binding site, with important conserved residues shown in RPP1^TIR^ (PDB: 7DFV). **c** The tetrameric assembly of TIR domains atop the NB-ARC domain of the RPP1 resistosome, and the positions of key interfaces and regions (PDB: 7CRC, 7DFV). **d** The DE surface, with important conserved residues shown in RPP1^TIR^ (PDB: 7DFV). **e** Proposed assembly of the SARM1^TIR^ octamer (Figley et al. 2021), based on symmetry within the SARM1^TIR^ crystal structure (PDB: 6O0R). **f** Comparison of the RPP1^TIR^ tetramer size (PDB: 7DFV) to the NLRC4 inflammasome (white, PDB: 3JBL). It is not known how plant TIRs would assemble on top of the inflammasome formed by the RPS4^TIR^:NLRC4 fusion protein (Duxbury et al. 2020)

In terms of self-association, an “AE interface”, involving residues within the αA and αE helices, has been repeatedly found in the crystal structures of AtTIR (Chan et al. 2010), RPS4^TIR^ and RRS1^TIR^ hetero- and homodimers (Williams et al. 2014), RPV1^TIR^ (Williams et al. 2016), SNC1^TIR^ and RPP1^TIR^ (Zhang et al. 2017a). The defining feature of the AE interface is an intercalating histidine core, surrounded by interactions between residues on the αA and αE helices of both TIR monomers (Fig. 5a). Residues in this interface are also highly conserved, and mutations to the AE interface impair the ability of the RPS4^TIR^, many other plant TIR domains and full-length TNLs, to self-associate and to induce auto-activity and effector-triggered HR (Mestre and Baulcombe 2006; Bernoux et al. 2011; Williams et al. 2014, 2016; Bentham et al. 2017; Zhang et al. 2017a; Ma et al. 2020; Martin et al. 2020).

The structure of RUN1^TIR^ was key in identifying the potential NAD^+^ binding site. RUN1^TIR^ was crystalized with Bis–Tris and NADP^+^ in the binding site, forming a dense network of interactions with many conserved residues (Horsefield et al. 2019). A glutamate at the base of the binding pocket is highly conserved (Fig. 5b). Mutagenesis to this residue abrogates innate immune signalling (Krasileva et al. 2010; Nishimura et al. 2017; Horsefield et al. 2019; Wan et al. 2019; Ma et al. 2020; Martin et al. 2020) and NADase activity (Horsefield et al. 2019; Wan et al. 2019), while not impairing the TIR domain’s ability to self-associate. Many of the residues involved in this NAD^+^ binding site are conserved in SARM1 (Horsefield et al. 2019).

ROQ1 and RPP1 resistosome structures explained the previous observations of AE and other interfaces in the crystals of plant TIR domains. In both tetrameric resistosomes, the TIR domains are arranged in a “dimer of dimers” atop the NB-ARC domains (Fig. 5c). The AE interface is the key interface, with two AE interfaces corresponding to the two dimers within the TIR tetramer. The other asymmetric interface has been termed the “BE interface”, which involves the BB-loop and a previously implicated “DE surface” involving the αD and αE helices (Zhang et al. 2017a) (Fig. 5c, d). BB-loop conformations are different in TIR domains between the active and inactive NLRs. In the active form, the BB-loop is tucked under the DE surface of the adjacent monomer, effectively creating a large NAD^+^ binding site between the BB-loop, DE surface and NAD^+^ binding site identified in RUN1^TIR^. Within the tetramers, there are, therefore, likely to be two binding sites (Fig. 5c). Residues within the NAD^+^ binding region are highly conserved, and mutations impair HR and NADase activity in different TIR domains (Dinesh-Kumar et al. 2000; Mestre and Baulcombe 2006; Swiderski et al. 2009; Krasileva et al. 2010; Bernoux et al. 2011; Williams et al. 2016; Nishimura et al. 2017).

Is the tetrameric arrangement seen in ROQ1 and RPP1 likely to be the same arrangement for all activated TNLs? Without more TNL structures, or other biochemical data, it is hard to predict. In the crystal structure, TIR domains of SARM1 arrange in an analogous fashion to ROQ1 and RPP1 tetramers (Fig. 5c, e). Assays with oligomeric chimaeras in planta provide some further clues. Horsefield et al. (2019) demonstrate that RPS4^TIR^ and RUN1^TIR^ domain fused to the central tandem SAM domains of human SARM1 could induce EDS1-dependent cell death in planta. The tandem SAM domains form a stable octamer in vivo and in vitro, and mutations that disrupt this octameric assembly also disrupt the activity of the SAM:plant TIR chimaeras. How plant TIR domains would arrange on top of the SAM octamer is unknown, but it may mimic the 2 × 4 arrangement proposed by the SARM1 octamers (Figley et al. 2021) (Fig. 5e).

Duxbury et al. (2020) fused RPS4^TIR^ to the NLRC4 protein, to test whether oligomerization induced by the chimeric protein activates TIR signalling in plants. NLRC4 forms an open-ended wheel-like oligomer, seeded in response to binding NAIP1, 2 or 5 and the activating ligand YscF, PrgJ or FlaA, respectively. When RPS4^TIR^ is fused to this oligomeric platform, it induces HR; however, the expected accumulation of v-cADPR was not detected, and resistance to *P. syringae* was not provided (Duxbury et al. 2020). This discrepancy could be explained by low levels of NADase activity displayed by plant TIR proteins (Horsefield et al. 2019). What arrangement the plant TIR domains would form atop this chimeric oligomer is also unclear (Fig. 5f), but it seems that this assembly is not sufficient for plant disease resistance. One hypothesis is that the assembly is sufficient to open the NAD^+^ active site for NAD^+^ hydrolysis, but not enough to complete the proposed binding site conformation seen in the BE interface of the ROQ1 and RPP1 structures. New structures of other TNLs will be required to determine if they can only form tetramers. Furthermore, there is evidence for heteromeric association of TNLs. RPS4 and RRS1 can form an inactive hetero-dimer (Williams et al. 2014). A study has also shown that the TIR domain of DM1 (dangerous mix 1) from a particular Arabidopsis accession can interact with that of DM2d (part of the *RPP1* complex locus), triggering auto-immune responses (Tran et al. 2017).

Apart from NADase activity, plant TIR domains were also found to function as 2′,3′-cAMP/cGMP synthetases by hydrolyzing DNA and RNA (Yu et al. 2021). This newly discovered enzymatic activity also contributes to cell death elicitation in plants, suggesting an essential role for the 2′,3′-cAMP/cGMP synthetases in TIR-mediated immune response (Yu et al. 2021).

## Products of the NAD^+^-cleavage reaction are required for downstream immune signalling

Upon the breakdown of NAD^+^, SARM1 cyclizes some of ADPR into cADPR (Essuman et al. 2017; Huang et al. 2019; Sasaki et al. 2020). While SARM1 has been proposed to trigger axon degeneration due to energetic failure upon rapid depletion of NAD^+^ (Gerdts et al. 2015; Yang et al. 2015), a recent study suggests that the cADPR produced by SARM1 causes calcium influx in neurons, which promotes axon degeneration (Li et al. 2021). On the other hand, NAD^+^ cleavage by a class of bacterial TIR domain-containing proteins results in formation of v-cADPR (Essuman et al. 2018). The v-cADPR produced by bacterial TIR domain-containing proteins has been found to activate another NAD^+^ consuming enzyme in response to phage infection (Ofir et al. 2021). Cyclic products generated by the animal and bacterial TIR domain-containing proteins have been proposed to act as the signalling molecule that causes cell demise.

NAD^+^ hydrolysis by plant TIR domains also leads to production of v-cADPR (Wan et al. 2019). A study showed that a v-cADPR producing bacterial TIR domain-containing protein (AbTir) did not cause any HR in planta when expressed as a chimeric NLRC4 protein (Duxbury et al. 2020). One possibility is that if cell-death signalling is mediated by the formation of a TNL signalling scaffold, bacterial TIR proteins may fail to serve as part of such a plant-specific complex. A more plausible scenario is that v-cADPR produced by AbTir could be slightly different from the plant-specific v-cADPR and thereby would fail to activate HR signalling. In accordance, TIR domain-containing proteins appear to produce several species of v-cADPR that have different effects. It was shown that v-cADPR produced by AbTir is different from that produced by HopAM1 (a *P. syringae* effector suppressing plant innate immunity) (Eastman et al. 2021). Interestingly, v-cADPR produced by a plant TIR domain-containing protein (BdTIR) was found to activate the bacterial antiphage defence system (Ofir et al. 2021). Alternatively, while AbTiR has NADase activity similar to some plant TIR proteins (Duxbury et al. 2020), it does not possess nuclease and 2′,3′-cAMP/cGMP synthetase activity shown to be important for plant cell death initiated by L7^TIR^ and RBA1 (Yu et al. 2021).

The NADase activity of the isolated plant TIR domain-containing proteins is quite low, compared to the isolated TIR domain of human SARM1 (Horsefield et al. 2019). Yet, the low catalytic activity is enough to activate cell death responses in an EDS1-dependent manner (Horsefield et al. 2019; Wan et al. 2019), and catalytically dead plant TIR mutants have no cell-death activity (Horsefield et al. 2019; Wan et al. 2019), which indicates that the NADase activity is quite crucial to the plant TIR-mediated HR responses. The role of v-cADPR in the plant immune system, however, remains unclear.

## Downstream pathways—the EDS1 family and helper RNLs control immune signalling activated by TNLs and some CNLs

TNLs and CNLs are sensor NLRs, as they directly or indirectly recognize effectors and require a network of downstream helper NLRs (Jubic et al. 2019; Feehan et al. 2020). RNLs including the ADR1 and NRG1 subfamilies constitute helper NLRs in angiosperms. RNLs are defined by their N-terminal CC_R_ domains with similarity to RPW8, as well as their unique NBDs (Chini and Loake 2005; Collier et al. 2011; Zhong and Cheng 2016). CC_R_ domains are reminiscent to four-helix bundle CC_HELO_ (HET-S/LOP-B) domains identified in fungal and animal membrane pore-forming proteins (Barragan et al. 2019; Huang et al. 2019; Feehan et al. 2020; Jacob et al. 2021). All studied plant genomes possess *RNL-*related gene families, suggesting their ancient origin (Shao et al. 2016; Zhong and Cheng 2016). RNLs diverged into ADR1 and NRG1 subfamilies, before the split between monocots and dicots (Collier et al. 2011). The repertoire of the RNL family members is limited, compared to vastly expanded of TNLs and CNLs (Shao et al. 2016). The Arabidopsis genome encodes three NRG1 paralogues (NRG1.1, NRG1.2 and NRG1.3) and four ADR1 paralogues (ADR1, ADR1-L1, ADR1-L2 and ADR1-L3) (Bonardi et al. 2011; Wu et al. 2019; Saile et al. 2020). RNLs (NRG1.1, NRG1.2, ADR1, ADR1-L1 and ADR1-L2) are required for both basal resistance and ETI activated by various TNLs and some CNLs (Bonardi et al. 2011; Dong et al. 2016; Jubic et al. 2019; Saile et al. 2020). Both the ADR1 and NRG1 subfamilies contribute to ETI responses, such as pathogen disease resistance, transcriptional reprogramming and HR, with an additional function of ADR1 proteins in basal resistance (Saile et al. 2020). Analyses of Arabidopsis mutants deficient in helper RNLs have led to a conclusion that the two RNL subclasses can similarly or differentially contribute to common functions (Bonardi et al. 2011; Dong et al. 2016; Castel et al. 2019; Lapin et al. 2019; Wu et al. 2019; Saile et al. 2020). The degree of RNL functional redundancy and specialization varies depending on sensor NLRs (Saile et al. 2020). For example, many tested TNLs, including RPS4:RRS1, RPP2, WRR4A, SNC1 and SOC3:CHS1, require contributions from both NRG1 and ADR1 family proteins (Dong et al. 2016; Wu et al. 2019; Saile et al. 2020). On the other hand, ETI initiated by RPP4 and CNLs, such as RPS2 and RPS5, is mostly dependent on ADR1 family members (Dong et al. 2016; Castel et al. 2019; Wu et al. 2019; Sun et al. 2021). Additionally, NRG1.3, which lacks the CC_R_ domain and is not directly involved in disease resistance (Wu et al. 2019), may act as a negative regulator of defence signalling through interaction with the EDS1 family members (Sun et al. 2021).

Another key component of NLR-activated host cell death and disease resistance is the EDS1 (enhanced disease susceptibility 1) family, consisting of EDS1, PAD4 (phytoalexin deficient 4) and SAG101 (senescence-associated gene 101) (Parker et al. 1996; Feys et al. 2001, 2005). The EDS1 family members are non-NLR proteins found only in seed plants (Wagner et al. 2013; Lapin et al. 2019) and share an N-terminal lipase-like α/β-hydrolase fold domain and unique C-terminal α-helical bundles named the EP (EDS1-PAD4) domain (Wagner et al. 2013). EDS1 family proteins do not possess enzymatic activity, despite the conserved catalytic residues (S-D-H) in the lipase-like domain (Wagner et al. 2013), but rather act as protein scaffolds. EDS1 forms heterodimeric complexes with SAG101 and with PAD4 (Wagner et al. 2013). The crystal structure of the EDS1:SAG101 complex (PDB: 4NFU) and modelling of the EDS1:PAD4 heterodimer demonstrate that their interaction is largely mediated by the N-terminal lipase-like domains, between the αH helix (LLIF) of EDS1 and a hydrophobic pocket of SAG101 or PAD4, as well as weak interactions contributed by their EP domains (Wagner et al. 2013). In general, the EDS1:SAG101 heterodimer preferentially contributes to TNL-dependent ETI responses such as HR and transcriptional regulation (Qi et al. 2018; Gantner et al. 2019; Sun et al. 2021). On the other hand, the EDS1:PAD4 heterodimer broadly contributes to basal defences and ETI initiated by both TNLs and CNLs, by enforcing salicylic acid biosynthesis (SA) and working in parallel with the SA signalling pathway (Cui et al. 2017). EDS1:PAD4-dependent transcriptional reprogramming, induced by activated TNLs or CNLs (RPS2), requires R493 of EDS1, which is located within a region at the EP surface involving multiple positively charged Lys, Arg and His residues (Bhandari et al. 2019). How EDS1 can integrate signals from TIR or CC domains remains an open question.

It has become increasingly evident that in angiosperms, the EDS1 family has evolved to function cooperatively with helper RNLs, by forming distinct signalling modules of EDS1:PAD4:ADR1 and EDS1:SAG101:NRG1 (Lapin et al. 2019; Wu et al. 2019, 2021; Sun et al. 2021). This view is indirectly supported by phylogenetic observations of co-occurrence of ADR1 and PAD4 and NRG1 and SAG101 orthologs in specific lineages. For instance, NRG1 and SAG101 orthologs are present in most angiosperms except Caryophyllales and monocots, and both families are absent in gymnosperms (Collier et al. 2011; Lapin et al. 2019). The Arabidopsis *pad4* single and *adr1* triple mutants are similarly more susceptible to pathogens, compared to wild-type plants, and their phenotype is similar in combined knockouts of *pad4* and *adr1s* (Sun et al. 2021; Wu et al. 2021), suggesting that these two components operate in the same pathway. Similarly, *sag101 nrg1* triple mutants phenocopy *sag101* single or *nrg1* double mutants, displaying enhanced susceptibility upon activation of the TNL pair RPS4:RRS1 in Arabidopsis (Sun et al. 2021; Wu et al. 2021). Exchanging components of the EDS1:RNL combinations or knockout of all RNLs result in further increased susceptibility to pathogens (Sun et al. 2021; Wu et al. 2021). These results suggest that the integrity of each intact module is required; the two branches can operate in parallel pathways, differentially contributing to common functions; and they may also have compensatory functions when either module is missing. It appears that each functional EDS1-RNL module preferentially elicits specific immune outputs, depending on sensor NLRs as mentioned above (Saile et al. 2020). Furthermore, variations in immune contribution by the two sectors most likely exist across different species (Lapin et al. 2019). In Arabidopsis and *N. benthamiana*, TNL-induced cell death predominantly relies on the EDS1:SAG101:NRG1 branch (Peart et al. 2005; Qi et al. 2010; Lapin et al. 2019; Wu et al. 2019). On the other hand, the Arabidopsis EDS1:PAD4:ADR1 module has a major role in basal immunity, including pathogen growth suppression, while its contribution to HR is seemingly limited (Lapin et al. 2020). Further research is required to establish whether mechanisms for such specific immune contribution require precise regulation of each complex involving spatial and temporal activation (Lapin et al. 2020).

## TNL-mediated immune signalling

**Fig. 6:**
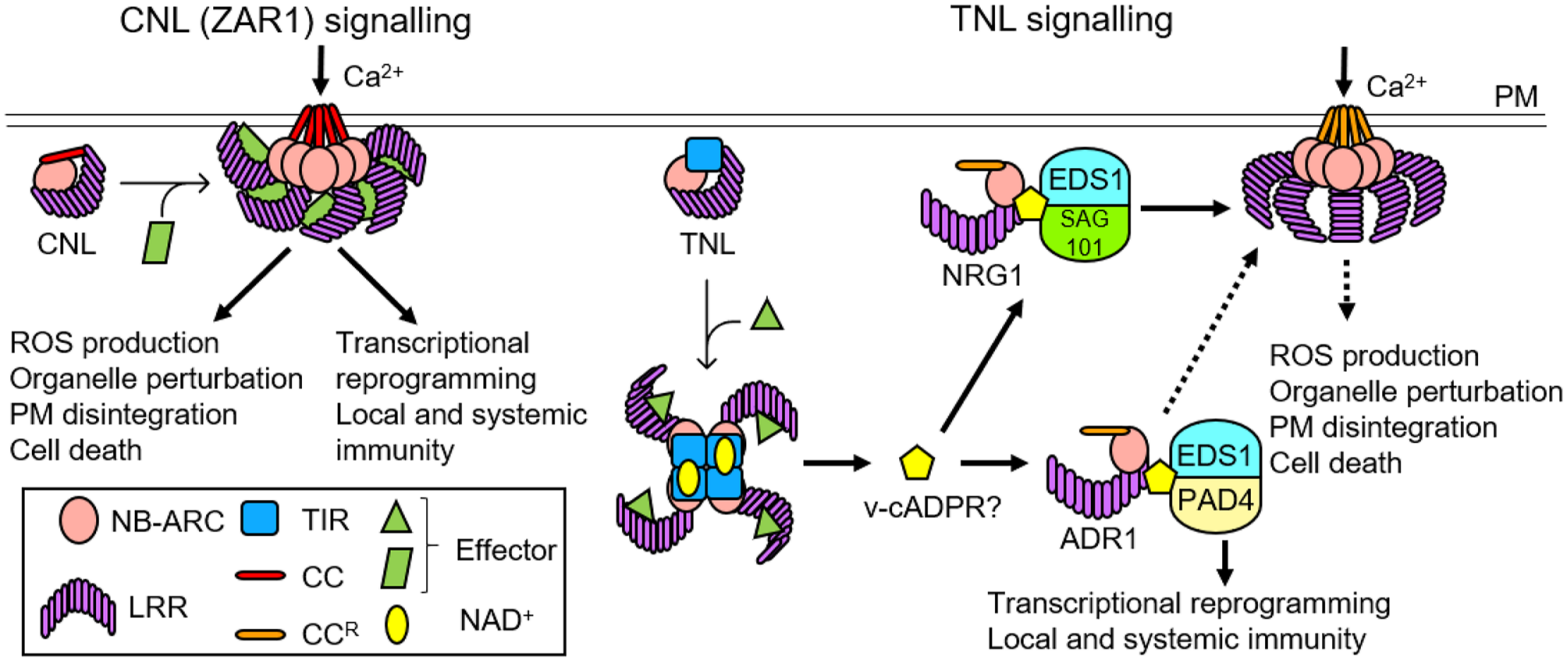
Working model of NLR-mediated plant innate immune signalling. In a resting state, CNLs and TNLs are in monomeric forms. Upon pathogen recognition, some CNLs, such as ZAR1, form resistosomes that insert into the plasma membrane (PM) and likely act as calcium-permeable channels (Bi et al. 2021). Activated TNLs form resistosomes that act as NAD^+^-cleaving enzymes (Ma et al. 2020; Martin et al. 2020). NAD^+^ hydrolysis results in production of plant-specific product, v-cADPR (Wan et al. 2019), which is proposed to signal through downstream components, the EDS1 family and RNLs. TNL activation induces formation of two distinct modules of EDS1:RNL complexes: (1) EDS1:SAG101:NRG1 and (2) EDS1:PAD4:ADR1 (Sun et al. 2021; Wu et al. 2021). Activated RNLs can oligomerize at the plasma membrane and form calcium-permeable channels (Jacob et al. 2021). Calcium influx triggered by the CNL and RNLs is suggested to induce oxidative burst, perturbation of organelles, disintegration of the cell membrane and eventual cell death (Bi et al. 2021). In addition, the EDS1:PAD4:ADR1 module activates transcriptional reprogramming, leading to basal innate immune responses

The EDS1 heterodimers provide a key link between sensor and helper NLRs, likely by forming a scaffolding platform (Lapin et al. 2020). It is known that some TNLs (SNC1, RPS4, RPS6, VICTR) directly interact with EDS1 or PAD4 in the nucleus or cytoplasm, consistent with the nuclear localization of the EDS1:SAG101 complex and nucleocytoplasmic localization of the EDS1:PAD4 complex (Feys et al. 2005; Bhattacharjee et al. 2011; Heidrich et al. 2011; Kim et al. 2012). Hence, it is possible that upon pathogen recognition, oligomerized TNLs may specifically recruit the EDS1 heterodimers. No direct protein–protein interaction has been reported between TNLs and RNLs (Lapin et al. 2020). In addition to genetic evidence for the two modules mentioned before, recent studies have provided the first evidence that the Arabidopsis EDS1:SAG101 and EDS1:PAD4 complexes exclusively interact with NRG1s and ADR1s, respectively, upon effector-TNL binding (Sun et al. 2021). The size of the EDS1:SAG101:NRG1 containing complexes extracted from Arabidopsis nuclei ranged from 100 to 600 kDa (Sun et al. 2021). Another study demonstrated that the Arabidopsis TIR-only protein RBA1 strongly induces association between EDS1:PAD4 and ADR1, as well as self-association of ADR1, in a manner dependent on its NADase activity (Wu et al. 2021). Therefore, the current working model (Fig. 6) proposes that activated TNL resistosomes may directly associate with EDS1 heterodimers, and/or the products of NAD^+^ hydrolysis by TNL resistosomes are perceived as signalling molecules by the EDS1 heterodimers or RNLs to promote the formation of activated EDS1-RNL protein complexes (Sun et al. 2021; Wu et al. 2021). Oligomerized RNLs then form calcium influx channels to promote cell death (Jacob et al. 2021). Additionally, given that ADR1 is constantly localized to the plasma membrane (Saile et al. 2021), it is likely that it interacts with cytoplasmic EDS1:PAD4 complexes to activate ADR1-dependent calcium influx, consequently leading to appropriate immune responses, including ROS production, transcription reprogramming and cell death.

## TIR domain-containing proteins in flowering plants

Apart from prototypical TNLs, additional two families of TIR-containing proteins, termed TIR-unknown site (TX) and TIR-NBD (TN), have been described in the Arabidopsis Columbia-0 ecotype (Meyers et al. 2002). TN homologs are also found in monocots, basal angiosperms and magnoliids (Nandety et al. 2013), although the mechanisms of their function remain to be established. TN proteins are composed of a TIR domain and much of the NB domain, but lack the LRR, while TX proteins lack both NB and LRR domains entirely (Meyers et al. 2002). Certain *TX* and *TN* genes are induced upon application of defence phytohormones, SA or jasmonic acid and confer innate immune responses, including EDS1-dependent cell death, when overexpressed in *N. benthamiana* and Arabidopsis (Nandety et al. 2013). Arabidopsis TN8 and TN11 proteins mediate cold temperature-dependent immunity (Nasim et al. 2020). The TIR-only proteins RBA1, from Arabidopsis, and BdTIR, from a monocot that lacks SAG101 and NRG1 proteins, induce EDS1-dependent cell death along with the helper NLR, NRG1, in the heterologous *N. benthamiana* expression system (Wan et al. 2019). RBA1 and BdTIR are also able to cleave NAD^+^ into v-cADPR, which appears to occur upstream of EDS1 and NRG1 (Wan et al. 2019). Although the mechanism behind the cell death activity of other TN and TX proteins is yet to be studied, it is possible that they possess NAD^+^ cleaving properties similar to TNL proteins. Phylogenetic analyses of these atypical genes indicate a diversion from and co-evolution with typical TNL genes, with which they form complex gene clusters within the Arabidopsis genome (Meyers et al. 2002). These genetic associations suggest linked functions of these R protein families in innate immunity, and TX and TN proteins potentially interact with pathogen effector proteins as well as TNLs, based on yeast two-hybrid screening (Nandety et al. 2013). One such example is a gene cluster encoding TN1 (also known as CHS1), TN2 and a TNL named SOC3 (also known as WRR12), which confers resistance to *Albugo candida* (Cevik et al. 2019), tandemly arranged in a head-to-head direction on the Arabidopsis chromosome 1. Whereas wild-type CHS1 interacts with only NB and LRR of SOC3, mutations in *CHS1* lead to interaction with TIR, NB and LRR domains of SOC3, which results in the activation of defence responses at low temperatures (Zhang et al. 2017b). Additionally, SOC3 interacts with both TN1 and TN2 proteins, to regulate the presence of the E3 ligase SAUL1 (senescence-associated E3 ubiquitin ligase 1), which is involved in supressing premature senescence (Liang et al. 2019). The TN1-SOC3 pair monitors the lack of SAUL1, while the TN2-SOC3 pair monitors SAUL1 excess accumulation. Similarly, full-length TNL40 and TNL60, located in a cluster containing nine *TN* genes, associate with TN10. All three genes are co-regulated upon pathogen infection, indicating the formation of complexes functioning in plant immunity (Chen et al. 2021). Interestingly, TN proteins also appear to function in CNL-mediated immunity. Both TN13 and TN21 interact with a CNL, RPS5, and the TN13:RPS5 complex specifically confers resistance to the *P. syringae* DC3000 carrying effector avrPphB (Cai et al. 2021).

## CNL-mediated immune signalling

While some CNLs require EDS1 family members and RNLs, others (including ZAR1) are likely capable of autonomously triggering cell death without the need of downstream helper NLRs (Saile et al. 2020) and elicit cell death through their own channel activity (Lewis et al. 2013; Bi et al. 2021) (Fig. 6). In Solanaceae, diverse sensor NLRs (CNLs) form a complex network relying on helper NLRs, contributing to cell death and disease resistance (Wu et al. 2017). The α1 helix of ZAR1^CC^, which drives pore formation, has a conserved MADA motif, with the consensus sequence MADAxVSFxVxKLxxLLxxEx (Adachi et al. 2019a). The MADA motif is present in ~ 20% of CNLs in flowering plants analysed, including ZAR1 and helper NRC4, while it is missing in Solanaceae sensor CNLs (Adachi et al. 2019a). The MADA motif in NRC4 was shown to be required for triggering cell death (Adachi et al. 2019a), suggesting a conserved mechanism of action by certain CNLs that likely undergo a conformational switch to form funnel-shape resistosomes.

Analysis of the N-terminal CC domains of Arabidopsis CNLs classified CNLs into four groups (Wroblewski et al. 2018). Group A contains an RPW8 motif (RNLs), while groups B, C and D contain the typical CC domain with an EDVID motif, which is found in the α3 helix, with variations. Group B is predicted to have an additional two short β strands, and group C has an additional conserved sequence of ten polar amino acids preceding the EDVID motif. Further functional studies should establish whether distinct mechanisms of action exist by each CNL group. Interestingly, large-scale interactome studies have revealed that both RNLs and CNLs can form heteromeric interactions just through their N-terminal CC domains, with more than two-thirds interacting with multiple CNLs (Wroblewski et al. 2018). Therefore, a complex CNL network likely exists forming highly redundant functional associations among CNLs and RNLs.

## Conclusions and future directions

The structures of CNL and TNL resistosomes significantly expand our understanding of the activation mechanisms of plant NLRs. However, there are still important questions to be answered about the nature of NLR activation and the ETI pathways. So far, ZAR1, ROQ1 and RPP1 structures demonstrate that activated monomers, each bound to the activating ligand, form a resistosome, which is similar to how human APAF-1 monomers, bound to cytochrome c, form a wheel-like structure (Dorstyn et al. 2018). However, the mechanism of oligomerization for the NAIP2:NLRC4 inflammasome is different from that of APAF-1, where recognition of PrgJ by a single molecule of NAIP2 is sufficient to drive recruitment of inactive NLRC4 monomers that undergo a conformational change to form a ring-like complex (Hu et al. 2015; Zhang et al. 2015). Whether plant NLRs utilize the NAIP2:NLRC4-like mechanism remains unknown, but such a mechanism is plausible, considering the heteromeric complex formation involving paired NLRs with ligand sensing and signalling roles. Additionally, it is also possible that some plant NLRs may utilize a mechanism similar to animal NLRP3 (Andreeva et al. 2021). Post-translational modifications of animal NLRs during activation have become increasingly evident in recent years (Yang et al. 2017). In the case of the RRS1-R:RPS4 TNL pair, phosphorylation has been shown to regulate their activation (Guo et al. 2020). Further research is required to elucidate effects of different post-translational modifications on NLR regulation. Although it remains unclear how activated TNLs trigger the association between EDS1:SAG101 and NRG1 and between EDS1:PAD4 and ADR1, the products of NAD^+^ cleavage by TNLs are potential candidates that serve as signalling molecules for bringing an RNL into the EDS1 scaffold (Fig. 6). Whether these catalytic products directly or indirectly induce a conformational change in the EDS1 family proteins or helper RNLs remains to be established. While some CNLs, such as ZAR1, are seemingly able to induce HR on their own, other CNLs most likely require downstream EDS1 and helper NLR proteins. In this case, how can different TNLs and CNLs activate the same set of EDS1:RNL downstream components? Furthermore, two recent studies add a new perspective towards the plant innate immunity; both intracellular NLRs and cell-surface pattern recognition receptors are required to fully activate immune responses, suggesting convergent mechanisms between ETI and PTI pathways (Ngou et al. 2021; Yuan et al. 2021). How the two types of plant innate immune receptors cooperatively promote immune signalling is yet to be characterized.

## Data Availability

Not applicable.
